# Iodoform in Diphtheria

**Published:** 1883-03

**Authors:** 


### IODOFORM IN DIPHTHERIA.

Diphtheria is a constitutional disease—this is not denied any more; but whether it is constitutional from the very beginning is not only very doubtful, but contrary to all sound reasoning. In case micrococci form really the pathogenic cause, they are first seen on the place of primary local affection, and from here they migrate and affect the blood in a similar, only more rapid, manner as the syphilitic virus. But granted that the disease is first purely local, and effective local treatment should be followed by such results as prove that the constitutional affection has been prevented by early destruction of the primary, local, existing evil. Dr. J. Benzon, in Buccari (*Wiener Med. Wochschrift*, 35–82), and Dr. S. Korach in Cologne (*Deutch Med. Wochschrift*, 36–82) both have made experiments with the local treatment of diphtheria by iodoform. The former treated one man, two women, two girls, respectively aged 18 and 22, and a four-year-old child; and notwithstanding the cases were all of grave type, under the local treatment mentioned, they all ran a very favorable and rapid course. B. treated his cases as follows: He took a painter's brush, 1 ctm. wide, and dipped it into a finely-powdered iodoform till the points were all colored yellow. He then depressed the back of the tongue and applied the iodoform with the brush to the diphtheretic membranes. This procedure was repeated by B. in the beginning of the disease every two hours, about eight times during the day and six times during the night. With the exception of ice application to the neck, this formed the whole treatment.

He made use of this treatment under and by direction of Prof. Leichtenstern, the physician-in-chief of the Cologne City Hospital. For nearly a year all the patients in this institution suffering from diphtheria were treated with iodoform alone; cleansing injections with unmedicated water being the only local or internal medication used besides. First, iodoform triturated with amylum, was insufflated; then the dry powder alone was put by a brush upon the membranes. Later these methods were dropped, and instead of them iodoform with collodium (1 : 10) brushed six times daily over the diphtheritic exudations, the latter having each time been previously totally dried with a linen rag. Sometimes a solution of 2.5 in 25.0 sulph-ether, and 5.0 tolu balsam, was made use of. The splendid results gained—of 213 cases only one death by laryngeal diphtheria—should induce far more extensive trials with this drug.—*Medical and Surgical Reporter.*

